# Redshifting and Blueshifting of β82 Chromophores in the Phycocyanin Hexamer of *Porphyridium purpureum* Phycobilisomes Due to Linker Proteins

**DOI:** 10.3390/life12111833

**Published:** 2022-11-09

**Authors:** Hiroto Kikuchi

**Affiliations:** Department of Physics, Nippon Medical School, 1-7-1 Kyonan-cho, Musashino, Tokyo 180-0023, Japan; kikuchi@nms.ac.jp

**Keywords:** absorption wavelength, energy transfer, linker protein, photosynthesis, phycobiliprotein, phycobilisome, phycocyanin, phycocyanobilin, *Porphyridium purpureum*, structure and function

## Abstract

Phycobilisomes in cyanobacteria and red algae are large protein complexes that absorb light and transfer energy for use in photosynthesis. The light energy absorbed by chromophores binding to phycobiliproteins in the peripheral rods can be funneled to the core through chromophores at very high efficiency. The molecular mechanism of excitation energy transfer within a phycobilisome is an example of a higher and unique function in a living organism. However, the mechanism underlying the high efficiency remains unclear. Thus, this study was carried out as a step to resolve this mechanism theoretically. The three-dimensional structure of phycobilisomes containing the linker proteins of the red alga *Porphyridium purpureum* was determined by cryoelectron microscopy at 2.82 Å resolution in 2020. Using these data, the absorption wavelength of each β82 chromophore in the phycocyanin hexamer located next to the core was calculated using quantum chemical treatment, considering the electric effect from its surrounding phycocyanin proteins and two linker proteins. In addition to unaffected chromophores, chromophores that were redshifted and blueshifted under the electrical influence of the two linker proteins were found. Namely, the chromophore serving as the energy sink in the rod was determined.

## 1. Introduction

Phycobilisome (PBS) is a large peripheral light-harvesting protein complex present on the cytoplasmic side of the thylakoid membrane in cyanobacteria and red algae [1,2,3,4,5]. PBS is typically hemidiscoidal in shape, with three cylindrical cores and six rods radiating from the core. Their cylindrical cores and rods are composed of phycobiliproteins and linker proteins, which bind chromophores. The light energy absorbed by chromophores in the peripheral rods can be funneled to the core via chromophores at very high efficiency [5]. This molecular mechanism of excitation energy transfer within PBS is an example of a higher and unique function in a living organism. However, the mechanism underlying the high efficiency is unclear, although the vibronic treatment of chromophores in recent studies [6,7,8,9] might lead to the resolution of this mechanism.

Since the 1980s, X-ray crystallography has been used to reveal the three-dimensional structure of PBS but without the linker protein as part of the structure. Recently, however, several three-dimensional structures of total PBS, including linker proteins, have been determined using cryoelectronic microscopy [10,11,12,13]. The present study is conducted using these structural data (2.82 Å resolution) that include linker proteins from the red alga *Porphyridium purpureum* PBS by Ma et al. [11] and the corresponding described notation, with the exception that the chromophore name is used.

**Figure 1:**
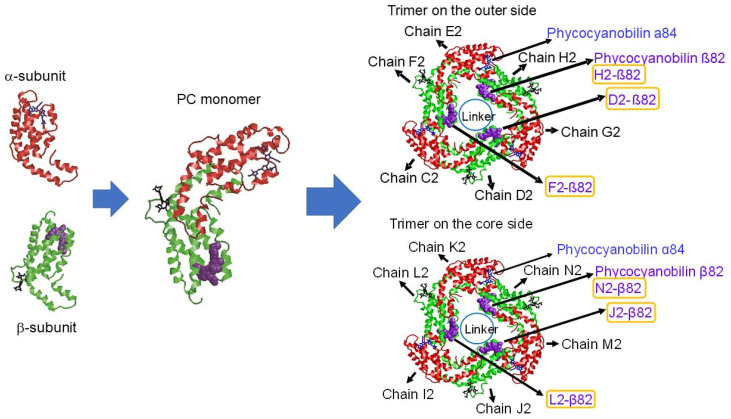
Schematic representation of α- and β-subunits, the phycocyanin (PC) monomer, and two PC trimers of the red alga *Porphyridium purpureum* phycobilisome (PBS). Proteins are depicted using cartoon representation. The chromophore bound to the 84th amino acid in the α-subunit (α84), the chromophore bound to the 82nd amino acid in the β-subunit (β82), and the chromophore bound to the 153th amino acid in the β-subunit (β153) are depicted using blue stick, purple spacefill, and black stick representations, respectively. The corresponding Protein Data Bank (PDB) ID for the structure is 6KGX.

Interestingly, PBS can be composed of several hierarchical structures accompanying the functions if we consider one protein chain as the smallest unit of the structure. An Asp residue in the globin fold of each phycocyanin (PC) subunit promotes the protonation of the chromophore, which confers light absorption properties [14,15,16,17,18,19,20]. The second hierarchical structure is the monomer (see Figure 1). The X–Y helix portion of the subunit causes an association between the α- and β-subunits and, at the same time, prevents fluctuations of the Asp residue, which stabilizes chromophore protonation [21,22]. The third hierarchical structure is the trimer, which is a basic block of PBS. The fourth hierarchical structure is a hexamer consisting of two trimers joined face to face. Bound to the outside of the PBS rod cylinder are chromophores that are not present on the outside of the PBS core cylinder. The hexameric structure modulates the optical absorption wavelength of the outside chromophore and effectively transfers the light energy absorbed at the outer chromophore to the inner chromophores [23]. In addition, light adaptation and chromatic acclimation processes in PBS function as hexamer units [24,25,26].

The fifth hierarchical structure, PBS, is created by the addition of a linker protein that connects the hexamers. In the PBS rod, the possibility of acquiring more light energy is increased by the connection of numerous hexamers. Thus, PBS is an antenna system that is established with a hierarchical structure, and its functions are also generated by the hierarchical structure, which requires true multiscale handling.

Linker proteins are present in the hollow portion of the trimeric or hexameric ring-shaped structure. In the case of the PC hexamer in *Porphyridium purpureum* PBS, six chromophores, called β82, are located where they face the hollow portion. The linker proteins have a structural role as linkers facilitating binding between the hexamers in the rod and between the PC hexamer and the core, but they are also expected to participate in the function of energy transfer. The trimers and hexamers have a symmetrical structure, and it is not possible to differentiate the optical absorption properties of the six β82s in the trimer. To ensure that energy transfer is efficient, the absorption wavelengths of these six β82s must differ, and the linker is expected to be responsible for this through electric interaction.

**Figure 2:**
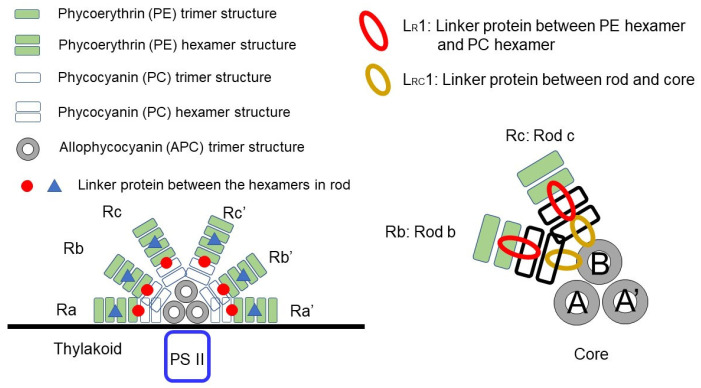
The schematic representation of a part of *Porphyridium purpureum* PBS depicted to explain the location of the PC trimer, the PC hexamer, the linker between the rod and core L_RC_1, and the linker between the PC hexamer and phycoerythrin hexamer (PE) L_R_1. In the lower-left corner, all rods, including the PC hexamer and all cores from *Porphyridium purpureum* PBS, are shown. In the lower right, it can be seen that rod b (Rb) and rod c (Rc) are bound to the top of core B. The notation A, A′, or B, written in the center of the cores, indicates the name of the core.

In this paper, we focus on the PC hexamer and the two linker proteins L_R_1 and L_RC_1 of rod b (Rb) (see Figure 2). Based on the structural data obtained by Ma et al. [11], the absorption wavelength of each β82 chromophore in the PC hexamer located next to the top of core B was calculated using quantum chemical treatment, including the electric effect from its surrounding phycocyanin proteins and two linker proteins. When the two linker proteins were included, redshifted, blueshifted, and unaffected chromophores were observed. It also became apparent that the β82 with the lowest first excited state, namely, the energy sink, was found in the rod and was the β82 closest to the core. At the same time, we identified the respective linker portion that was responsible for β82 redshifting and blueshifting or that otherwise had no effect. This means that we clarified where and how much electric charge is required around the chromophore to achieve redshift or blueshift.

## 2. Materials and Methods

### 2.1. Data Used in This Study

The cryoelectron microscopy structure of a 14.7-megadalton phycobilisome (PBS) with a hemiellipsoidal shape from the red alga *Porphyridium purpureum* was used. The resolution was 2.82 Å, and the file name in the Protein Data Bank is 6KGX [11].

### 2.2. Net Charges and Hydrogen Coordinates

Before calculating the wavelength of light absorption, the hydrogen atoms were coordinated. For aspartic acid and glutamic acid, the hydrogen atom at the end of the side chain was removed to give a −1 charge, and for arginine and lysine, a hydrogen atom was added to the end of the side chain to give a +1 charge. First, three successive amino acid residues in α-subunits in the PC hexamer, β-subunits in the PC hexamer, L_RC_1, and L_R_1 of 6KGX were treated as a trimer, to which hydrogen atoms were added. Then, the coordinates of the hydrogen atoms were optimized with respect to the total energy of the trimer by using the Modified Neglect of Diatomic Overlap-Parametric Method 3 (MNDO-PM3) molecular orbital (MO) method [27,28]. For the trimer, the N-terminus was set to -NH_2_, but the C-terminus of the trimer, except for the C-terminus of α-subunits in the PC hexamer, β-subunits in the PC hexamer, L_RC_1, and L_R_1, was replaced with –COCH_3_ because the oxygen atom in –OH strongly attracts electrons. At the C-terminus of α-subunits in the PC hexamer, β-subunits in the PC hexamer, L_RC_1, and L_R_1, –COOH was used. The coordinates and net charges of the central amino acid residue in the trimer were selected and used except at both ends of the protein chains. These procedures were performed until the coordinates and net charges of all the amino acids in α-subunits in the PC hexamer, β-subunits in the PC hexamer, L_RC_1, and L_R_1 were obtained. These values were then utilized to determine the wavelength of light absorption.

The electrostatic interaction between these net charges and the electronic state of the chromophore β82 was considered a perturbation. As for the chromophores, hydrogen atoms were also added, and their geometry was optimized using the MNDO-PM3 MO method. Note, however, that not only the nitrogen atom on the B-ring of the chromophore but also the nitrogen atom on the C-ring was protonated, and the net charge on the whole chromophore was +1 [14,15,16,17,18,19,20].

### 2.3. Calculation of the Wavelength of Light Absorption and the Oscillator Strength

The wavelength of light absorption and the oscillator strength were calculated using the unique intermediate neglect of differential overlap-configuration interaction (INDO-CI) method [29]. All molecular integrals in the calculation were estimated as functions of electron densities of individual atoms according to Sakuranaga et al. [29]; this INDO-CI method differs slightly from that used by Pople et al. [30]. The resonance integrals were expressed using parameter *k*_β_ in Equation 2.2 in [26], as described by Wolfsberg and Helmholtz [31]. In the calculation of this research, all the values except for those of k_β_ are the same as those reported by Sakuranaga et al. [29]. The values of k_β_ were k_β_(C–C) = k_β_(C–O) = 1.10, k_β_(C–N) = 0.70, and k_β_(others) = 1.20 to reproduce the observed light absorption and oscillator strength of the α84 chromophore in Cyanophyta phycocyanin (C-PC) [14]. In addition, these parameters were used in the study of [23] and were the values that reliably reproduced the experimental results. Each chromophore was treated in the protonated form [14,15,16,17,18,19,20].

A total of 338 excited configuration interactions (CI), consisting of the lowest singly excited configurations, *ψ* (*j*, *m*), and doubly excited configurations, *ψ* (*jj*, *mm*), were considered as the wave function. In this work, *ψ* (*j*, *m*) was constructed by exciting an electron from an occupied molecular orbital (MO *ϕ _j_*) to an unoccupied molecular orbital (MO *ϕ _m_*) and *ψ* (*jj*, *mm*) by exciting a pair of electrons from MO *ϕ _j_* to MO *ϕ _m_*. For CI calculations, the interaction between the chromophore and its surrounding protein portions was considered the electrostatic interaction between the electronic states of the chromophore and the net charges of atoms in the surrounding protein portions. The number of CIs considered in this system has already been verified as appropriate in [23].

## 3. Results

**Figure 3:**
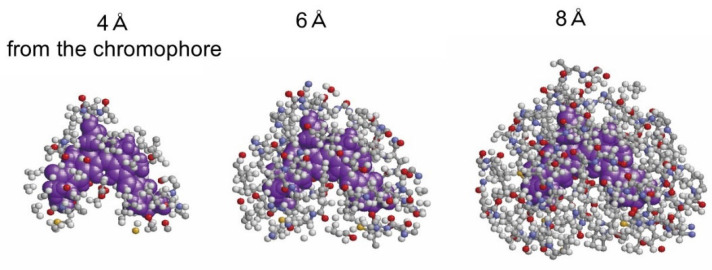
The chromophore β82 in F2, which is the original chain ID in [11], and the atoms of its environment within 4, 6, or 8 Å from β82. β82 is depicted by a purple spacefill representation and the other surrounding atoms by a ball-and-stick representation.

The chromophore geometry in the phycobilisome (PBS) is the chief factor that determines its electronic state. The second factor is the protonation of the chromophore [14,15,16,17,18,19,20], and the third is the electrical interaction in the environment. In this study, the electrical effect of the environment, consisting of 12 phycocyanin (PC) chains and two linkers L_R_1 and L_RC_1, is considered the function of the distance from the chromophore when the electronic state of each chromophore and the absorption wavelength λ_1_ of the S_0_ → S_1_ transition are calculated. Figure 3 shows the atoms of the environment within 4, 6, or 8 Å from β82 in F2, which is the original chain ID in [11].

**Figure 4:**
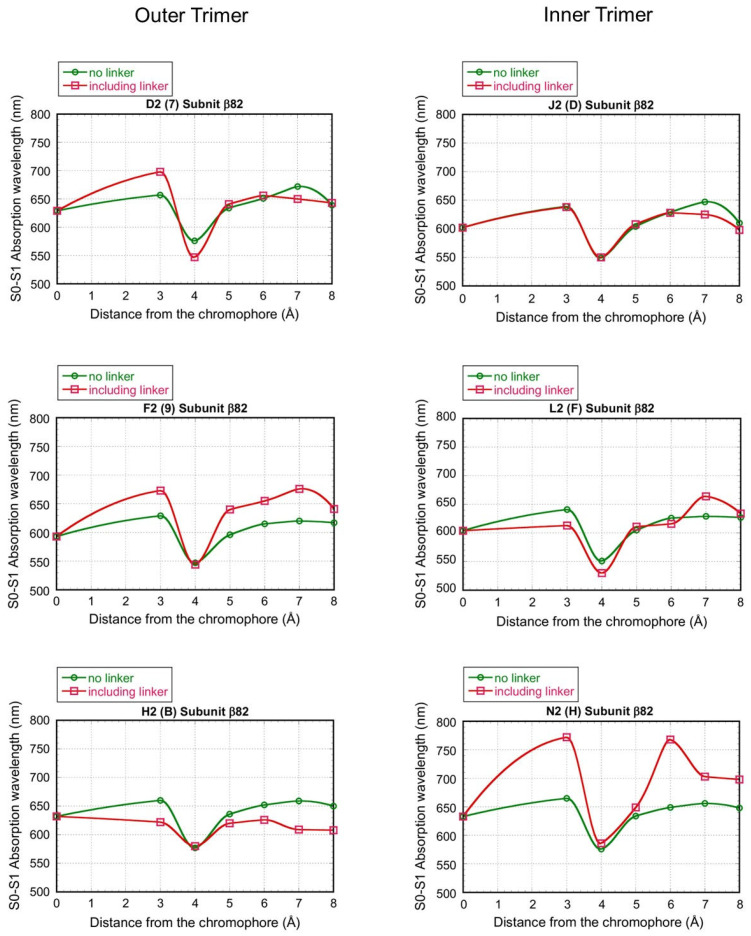
Dependence of the absorption wavelength λ_1_ of the S_0_ → S_1_ transition on distance *R* (Å) from each β82 chromophore in the six chains D2, F2, H2, J2, L2, and N2 of the “rod b PC hexamer”, calculated by including the electrostatic interaction of β82 with its surroundings within *R* (Å). Here, *R* means the longest distance from a given atom of the chromophore, including its hydrogen atoms, to any atom of the surroundings, and the β82 surroundings are classified into two cases: with and without the L_R_1 and L_RC_1 linkers. The red line and the green line represent the case with the linkers and the case without the linkers, respectively. The notation 7, 9, B, D, F, or H in the brackets represents the chain name in the file “6kgx-pdb-bundle1.pdb” of 6KGX.

Figure 4 shows the dependence of the absorption wavelength λ_1_ of the S_0_ → S_1_ transition, including the electrostatic interaction effect on distance *R* (Å) from each β82 chromophore in six chains D2, F2, H2, J2, L2, and N2 of the “rod b PC hexamer”. Here, *R* from the chromophore means the longest distance from a given atom of the chromophore, including its hydrogen atoms, to any atom of the surroundings. The names D2, F2, H2, J2, L2, and N2 represent the original chain ID in [11], and “rod b PC hexamer” means the PC hexamer in rod b (see Figure 2). In these calculations, the electrostatic interaction between each β82 chromophore and its surrounding atoms was classified into the following two cases and considered to investigate the effect of the linker proteins L_R_1 and L_RC_1: (1) the electrostatic interaction of the β82 chromophore with the surrounding atoms of PC and the linkers L_R_1 and L_RC_1 within *R* (Å) and (2) the electrostatic interaction of the β82 chromophore with the surrounding atoms of PC only (without the linkers) within *R* (Å). The red line and the green line in the graphs of Figure 4 show the results of cases 1 and 2, respectively. In addition, the chains J2, L2, and N2 are in the inner trimer (the nearer side to the core) of the PC hexamer, and the chains D2, F2, and H2 are in the outer trimer (the opposite side of the core) of the PC hexamer.

In the absence of influence from the surrounding environment, the chromophores are divided based on absorption maxima into two groups of F2, J2, and L2, with absorption maxima at around 600 nm, and D2, H2, and N2, with absorption maxima at around 630 nm. The maxima are determined by the geometrical structure of the chromophores themselves. If we consider the effect of the PC alone as the surrounding environment, i.e., ignoring the effect of the linker, we can similarly distinguish these two cases: the F2, J2, and L2 types and the D2, H2, and N2 types (see the green line). This is due to a break in the three-fold symmetry structure of the PC hexamer, which is also thought to determine the geometrical structure of the chromophore β82.

In the region where the value of R is small, the absorption maximum wavelength is unstable and fluctuates, but as the value of R increases, the fluctuation range becomes smaller and settles at a constant value.

The most significant feature is that the β82 of the N2 chain shows a large redshift due to the influence of the linker surrounding its β82, and the β82 in the H2 chain shows a blueshift due to the influence of the linker surrounding its β82. The other four β82s show maximum absorption wavelengths between 600 and 650 nm and are not as affected by the linker as the N2 and H2 chromophores.

**Figure 5:**
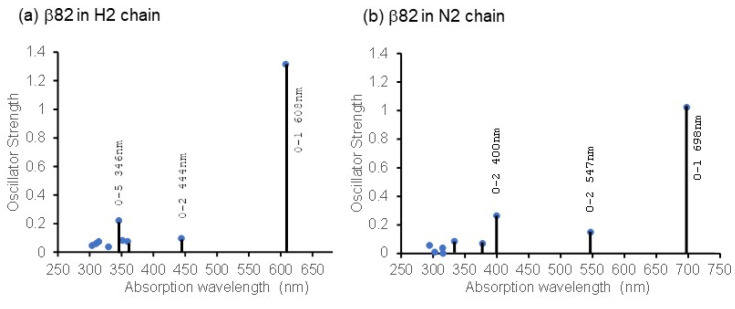
Optical absorptions of β82 chromophores in the H2 chain and the N2 chain. The vertical axis represents oscillator strength, and the horizontal axis represents absorption wavelength.

Figure 5 shows the calculated absorption wavelength of β82 in the H2 and N2 chains, considering the electrostatic interaction within 8 Å from the respective chromophores. It shows optical absorption characteristics that correspond with the experimental facts, such that only the S_0_–S_1_ oscillator strength is strongly represented, indicating the validity of the calculation.

## 4. Discussion

In this study, we have investigated how the six β82 chromophores of the phycocyanin (PC) hexamer adjacent to the core of the phycobilisome (PBS) are affected by two linker proteins, L_R_1 and L_RC_1, focusing on their optical absorption wavelength. The results reveal that β82 of the N2 chain in the PC hexamer is strongly affected by L_RC_1, resulting in a large redshift. This means that β82 of the N2 chain has the lowest excitation energy in the rod, i.e., it is the energy sink of all the chromophores of the rod.

**Figure 6:**
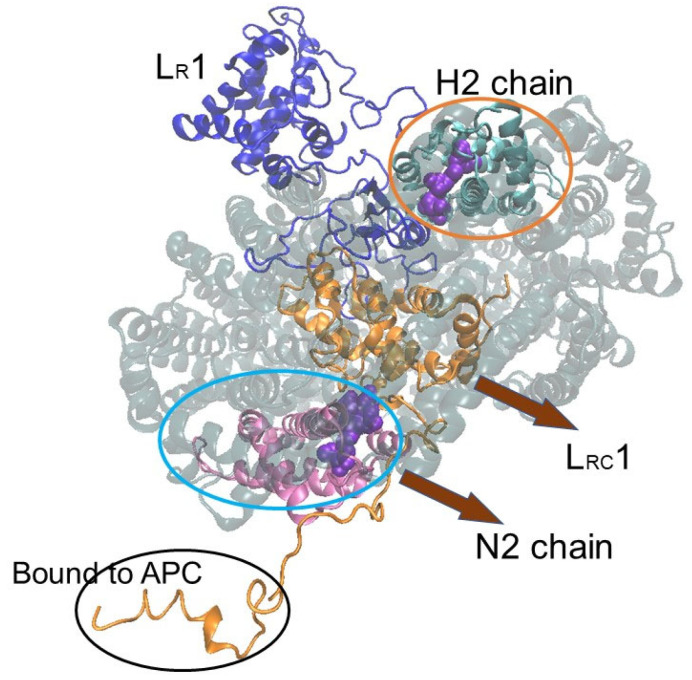
The location of the N2 chain, the H2 chain, L_R_1, and L_RC_1 in and around the phycocyanin (PC) hexamer. The N2 chain, the H2 chain, L_R_1, and L_RC_1 are shown in mauve, cyan, blue, and orange, respectively. The β82s in the H2 and N2 chains are depicted by a purple spacefill representation. The other four chains in the PC hexamer are shaded in gray. The terminal end of L_RC_1 binds to the core protein allophycocyanin (APC).

The distance from β82 to the two linkers L_R_1 and L_RC_1 indicates that L_R_1 affects the outer trimer of the PC hexamer but has little effect on the inner trimer. On the other hand, L_RC_1 affects not only the inner trimer but also the outer trimer slightly. The β82 in the N2 chain is not influenced by L_R_1 because it is far away from L_R_1, but it is influenced by L_RC_1 and is the closest chain to the core. This is a reasonable result when considering energy transfer. To illustrate this, we have depicted the PC hexamer with L_R_1 and L_RC_1 in Figure 6. The H2 chain, influenced by the linker L_R_1, shows a blueshift compared with the surrounding protein alone. It is also interesting that the β82 of the H2 chain is located furthest from the core in the PC hexamer in Figure 6.

Some might argue that semi-empirical MO methods are not sufficient. In fact, although it is difficult to obtain very high precision values with semi-empirical MO methods, it is possible to use the values obtained as indicators for useful discussions. In this work, the parameters of the INDO-CI method were set to reproduce the experimental values for the optical absorption wavelengths of α84 in C-phycocyanin [14], and these parameters were applied to calculate the absorption wavelengths of the β82s in this research. In order to reproduce the experimental value of the light absorption wavelength of β82, it may be necessary to change the parameter values slightly. However, this would not affect the content of the large redshift of β82 in the N2 chain or the conclusions of this study. Thus, this discussion, in terms of the calculation results using the INDO-CI method, is effective.

Some might also argue that the MNDO-PM3 MO method’s treatment of the proteins surrounding the chromophore is insufficient. Indeed, the MNDO-PM3 MO method may generally be inferior to ab initio or density functional theory (DFT) methods. However, keeping the same INDO parameters set to reproduce the wavelengths of α84 in C-PC [14] and setting the net charges of the oxygen and hydrogen atoms of water molecules as −0.3307 and 0.1653, respectively, obtained by the ab initio MO method for a water molecule at the HF/STO-3G level, with the net charges of the atoms constituting the protein calculated using the same method as in the present study, the observed S_0_–S_1_ absorption wavelength of the β155 chromophore of C-PC was successfully reproduced [23]. In the results of this study, the average value of the calculated S_0_–S_1_ absorption wavelength of the β82s in the PC hexamer, except for the β82 in the N2 chain, is about 620 nm when the atoms within the distance 8 Å from the chromophore are considered (Figure 4). This value could be said to reproduce the optical absorption wavelength properties described in [11]. Accordingly, it is unlikely that this approach will lead to the wrong conclusions in this study.

In this study, the factors that promote the redshift and blueshift of the chromophore β82 should also be noted. If the geometric structure of phycocyanobilin is the same as the β82 of the PC hexamer of red alga *Porphyridium purpureum*, phycocyanobilin can be redshifted or blueshifted by applying the same electrical influence as these linkers. Information on the linkers around β82 is useful when considering the physical properties of the chromophore phycocyanobilin. In particular, the influence from the surroundings within 6 Å from the β82 of the N2 chain showed a very large redshift (see Figure 4).

**Figure 7:**
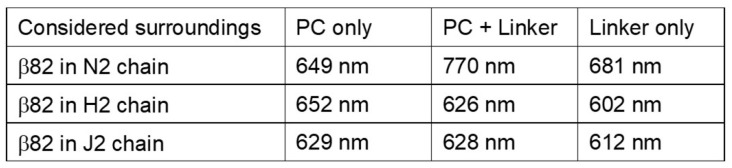
The effect of the surroundings within 6 Å from the β82 on the absorption maximum wavelength of the β82 of the N2 chain, the H2 chain, and the J2 chain in the following three cases: (1) when only PC atoms within 6 Å from the β82 are considered, (2) when all PC and linker atoms within 6 Å from the β82 are considered, and (3) when only linker atoms within 6 Å from the β82 are considered.

Figure 7 shows the effect of the surroundings within 6 Å from β82 on the absorption maximum wavelength of the β82 of the N2 chain, the H2 chain, and the J2 chain in the following three cases: (1) when only PC atoms within 6 Å are considered, (2) when all PC and linker atoms within 6 Å are considered, and (3) when only linker atoms within 6 Å are considered.

This shows that in the case of the β82 in the N2 chain, the effect from the linker alone also shows a blueshift, but when the PC hexamer is also included, the redshift is much larger; in the case of the β82 in the H2 chain, the effect from the linker alone shows a large blueshift, but the effect is weaker when the PC hexamer is also included. In the case of the β82 of the J2 chain, the effect from the PC hexamer results in a slight redshift, but the addition of the linker has no effect on the redshift. From these results, the effect of the linker L_RC_1 leading to the redshifting of the β82 of the N2 chain, the effect of the linker L_R_1 leading to the blueshifting of the β82 of the H2 chain, and the cases where the linker does not cause redshifting or blueshifting are further highlighted.

**Figure 8:**
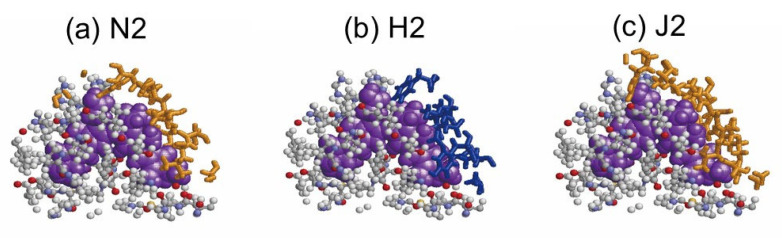
The linker portion causing redshifting and blueshifting and not causing any redshifting or blueshifting. These linker portions are depicted in a stick representation. (**a**) The linker L_RC_1 (orange), the β82 (purple), and the PC hexamer (ball and stick) within 6 Å from the β82 in the N2 chain, (**b**) the linker L_R_1 (blue), the β82 (purple), and the PC hexamer (ball and stick) within 6 Å from the β82 in the H2 chain, and (**c**) the linker L_RC_1 (orange), the β82 (purple), and the PC hexamer (ball and stick) within 6 Å from the β82 in the J2 chain.

Figure 8 shows the linker (L_RC_1) portion causing the redshift for β82 of the N2 chain, the linker (L_R_1) portion causing the blueshift for β82 of the H2 chain, and when the linker portion (L_RC_1) does not affect β82 of the J2 chain. In the three cases, different portions of the linkers are present at the same location around the β82 chromophore, potentially altering the optical absorption properties of β82.

**Figure 9:**
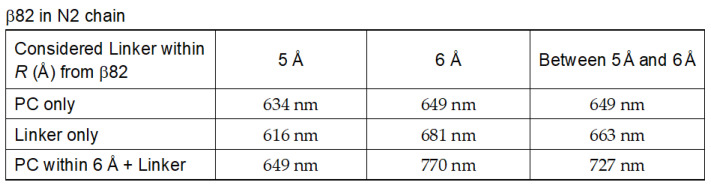
The S_0_–S_1_ absorption wavelengths considering the linker moiety within 5 Å, within 6 Å, and only between 5 and 6 Å from the chromophore for the cases of PC only, Linker only, and PC within 6 Å + Linker.

Additionally, we discuss the absorption maximum wavelength of the β82 of the N2 chain in more detail, focusing on the presence of a particularly special amino acid residue that leads to the redshift of the β82 of the N2 chain. Figure 9 describes the S_0_–S_1_ absorption wavelengths considering the linker moiety within 5 Å, within 6 Å, and only between 5 Å and 6 Å from the chromophore for the cases of PC only, Linker only, and PC within 6 Å + Linker.

With respect to the case where only the PC protein is considered an effect of the environment, the case where up to 6 Å is considered shows a slightly larger redshift than the case where up to 5 Å is considered. These values, namely, 649 and 634 nm, are not specific to the N2 chain. β82 shows a redshift, and its wavelength is 663 nm when only the linker portion from 5 Å to 6 Å is considered from β82 as an effect of the environment. Let this result be Result (i). The β82 of the case where up to 6 Å is considered shows a larger redshift than Result (i), and its wavelength is 681 nm. Let this result be Result (ii).

When both the PC protein portion within 6 Å from β82 and the linker from 5 Å up to 6 Å from β82 are considered, β82 has a wavelength of 727 nm and shows a larger redshift than Result (i). When both the PC protein portion within 6 Å from β82 and the linker within 6 Å from β82 are considered, β82 has a wavelength of 770 nm and shows a larger redshift than Result (ii).

The important point here is that the values of 727 and 770 nm are significantly larger than Results (i) and (ii). This implies that the interaction of the linker with the PC protein is more important rather than the presence of a special amino acid residue that alone causes the redshift between 5 and 6 Å from the β82 on the linker. Although there may be some key amino acids (especially charged amino acids) that lead to the redshift on the linker side, the effect of the amino acid alone is not responsible for the large redshift.

In this study, we found that the linker proteins L_RC_1 and L_R_1 affect the optical absorption properties, but they may also play a role in increasing the efficiency of energy transfer through other mechanisms.

## 5. Conclusions

According to the quantum chemical calculations of this research, the β82 chromophore in the N2 chain, which is the closest of those in the phycocyanin (PC) hexamer to the core, is significantly redshifted by L_RC_1, indicating that it is the energy sink of the rod. The β82 of the H2 chain farthest from the core in the PC hexamer is blueshifted by L_R_1. The results show that the excitation energy of the chromophore becomes smaller as it becomes closer to the core, which would be reasonable to express the energy transfer.

When the phycobilisome (PBS) is considered a protein aggregate or hierarchical structure, the linker protein, which links the hierarchy of hexamers to form a higher hierarchical structure, PBS, induces a change in the optical absorption wavelength properties of the smallest hierarchical unit. Using theoretical calculations, this study shows that a new and beneficial function is generated by the creation of a higher hierarchical structure, which is characteristic of the phenomenon of life.

## Data Availability

Not applicable.
